# Assessing Tissue Fixation Time and Quality with Label-free Mid Infrared Spectroscopy and Machine Learning

**DOI:** 10.1089/bio.2022.0108

**Published:** 2023-04-17

**Authors:** Daniel R. Bauer, David R. Chafin

**Affiliations:** Roche Diagnostics Solutions, Pathology Research and Early Development (Ventana Medical Systems, Inc.), Tucson, Arizona, USA.

**Keywords:** immunohistochemistry, raman spectroscopy, machine learning, predictive modeling, cancer diagnostics, tissue preanalytics

## Abstract

**Objectives:**

This work investigates whether changes in a biospecimen's molecular composition from formaldehyde fixation drive changes in the mid infrared (MID-IR) spectrum. Our ultimate goal was to develop an analytical metrology that could be used to accurately determine the fixation time of a tissue sample as a surrogate to overall tissue quality.

**Methods:**

Multiple unstained formalin-fixed paraffin-embedded tissue samples were scanned with an MID-IR microscope to identify a molecular fingerprint of formaldehyde fixation. The fixation specific patterns were then mined to develop a predictive model. A multiple tissue experiment using greater than 100 samples was designed to train the algorithm and validate the accuracy of predicting fixation status.

**Results:**

We present data that formaldehyde crosslinking results in alterations to multiple bands of the MID-IR spectra. The impact was most dramatic in the Amide I band, which is sensitive to the conformational state of proteins. The spectroscopic fixation signature was used to train a machine-learning model that could predict fixation time of unknown tissues with an average accuracy of 1.4 hours. Results were validated by histological stain quality for bcl-2, FOXP3, and ki-67. Further, two-dimensional imaging was used to visualize the spatial dependence of fixation, as demonstrated by multiple features in the tissue's vibrational spectra.

**Conclusions:**

This work demonstrates that it is possible to predict the fixation status of tissues for which the preanalytics are unknown. This novel capability could help standardize clinical tissue diagnostics and ensure every patient gets the absolutely best treatment based on the highest quality tissue sample.

## Introduction

Modern clinical histology is built on the 100+ year old principle of formaldehyde fixation that preserves a tissue's biostructure by arresting mechanisms of molecular degradation.^1^ Introduced by Ferdinand Blum, tissues are soaked in 10% neutral buffered formaldehyde (10% NBF) that slowly diffuses into tissue while forming covalent crosslinks between proteins and nucleic acids through primary amine groups. How thoroughly a tissue is fixed has a significant impact on antigen detection via modern immunohistochemistry (IHC) since most antibodies do not recognize native unfixed structures.^2–4^ The level of tissue fixation significantly impacts detected protein expression, which drives medical opinion on diagnosis, prognosis, and treatment.^5–11^

Once a biospecimen has been fixed and chemically crosslinked, its fixation cannot be altered and data regarding the total amount of formaldehyde exposure are often lost. However, paraffin tissue blocks are used for clinical trials for drug and product development. Non-standardized fixation protocols make it difficult to share data between medical facilities for similar antibody tests.^12^ Currently, clinical trials are performed by sourcing normal and diseased tissue blocks from biobanks where preanalytical data, such as fixation status, are often lacking. Given the importance of collecting reproducible and reliable data, medical research and routine clinical practice would benefit from techniques that could distinguish properly fixed samples from inadequately fixed samples.

There are currently no analytical methods for measuring formaldehyde exposure and fixation quality. Fixation quality typically is assessed through hematoxylin and eosin stains and analyzing cellular morphology. This qualitative examination is labor intensive, subjective, and capable of only distinguishing tissue quality in a coarse manner. Others have explored analytical techniques such as western blot and nuclear magnetic resonance imaging, but these are not compatible with routine histology nor have they been able to make a hard correlation with formaldehyde exposure and IHC quality.^13^ At present, no quantitative measure of formaldehyde exposure or crosslink formation is available.

Mid infrared (MID-IR) spectroscopy is a powerful non-destructive optical technique that probes the vibrational state of individual molecules in the tissue and is very sensitive to the conformational state of proteins.^14–16^ This extreme sensitivity makes MID-IR spectroscopy ideally suited for microscopy applications, because the conformational state of endogenous and exogenous materials manifest through changes in the MID-IR absorption profile of the biospecimen. Vibrational spectroscopy has been used for diagnostic applications, for example, to distinguish healthy from cancerous tissue.^17–23^

Tissue fixation is likely a complex synergy of chemistry and conformation changes, and we investigated whether these changes would manifest in alterations in the MID-IR spectrum. We used the MID-IR spectra, to develop a metrology that could be used to accurately determine the fixation time of a tissue sample as a surrogate to overall tissue quality. This novel capability will enable scientists and medical professionals to assess the fixation status of any tissue sample with a standardized and objective metrology.

## Methods

### Tissue collection and fixation

Human tonsil tissue was obtained fresh, unfixed, and de-identified from a local Tucson, AZ hospital under an approved contractual agreement. The human tonsil organs were medical waste, and therefore no IRB approval was required as the tissues were acquired under an exemption. Whole tonsils from same-day surgeries were transported to Roche Tissue Diagnostics (Ventana Medical Systems, Inc., Tucson, AZ) on wet ice in biohazard bags.

Individual 4 mm thick samples were procured from whole tonsil organs and differentially fixed for either 0, 1, 2, 4, 6, 12, or 24 hours in room temperature (typically 22°C–25°C) 10% NBF. Tissues from the original tonsil organs were randomly divided into groups of tissue fixation times. After fixation, samples were further processed in a Sakura ASP300 tissue processor (Sakura, Inc., Torrance, CA) set to an overnight cycle and embedded into a paraffin block.

In addition, whole unfixed human tonsil organs were fixed using a previously described two temperature cold+warm fixation method to purposefully prepare samples that had regions of fixed and under-fixed tissue.^24^ Whole unfixed tonsils were placed into 10% NBF (Saturated aqueous formaldehyde from Fisher Scientific, Houston, TX, buffered to pH 6.8–7.2 with 100 mM phosphate buffer) previously chilled to 4°C for 1, 2, 4, 17, 24, 72, and 168 hours. Samples were then placed into 45°C NBF for an additional 1 hour to initiate crosslinking. After cold+warm fixation, the whole organs were sectioned into 4 mm thick sections. The slices from the center of the organ, where the most variability would happen, were further processed and embedded into a paraffin block.

### Tissue staining

Immunohistochemical assays for bcl-2 (124) (no. 5986826001; VMSI), ki67 (30-9) (no. 5278384001; VMSI), and FOXP3 (SP22) (no. ab99963; Abcam) were performed on a Ventana Benchmark Ultra XT automated staining instrument according to the manufacturer's instructions and package inserts. Slides were de-paraffinized using EZ PREP solution (no. 5279771001; VMSI) at 72°C, and reagents and incubation times were chosen according to package inserts. Slides were developed using the OptiView DAB detection kit (no. 6396500001; VMSI) and counterstained with hematoxylin II (no. 5277965501; VMSI). All reagents were obtained from Ventana Medical Systems, Tucson, AZ.

### Brightfield slide imaging

The IHC-stained tissues on microscope slides were obtained from tissue specimens that were differentially fixed in 10% NBF. After cover-slipping, slides were scanned on a Ventana HT microscope slide scanner with a 20 × magnification. Files were stored as .bif files and analyzed using custom image processing software developed in MATLAB (Mathworks).

### Quantitative machine-learning model to predict fixation time

To develop a more accurate method of determining fixation time, and therefore quality of the tissue biospecimen, a machine-learning model was developed based on the partial least-squares regression (PLSR) algorithm, also known as projection on latent structure regression (PLSR). To train the algorithm, all 210 slides from the differentially fixed blocks were put into a database and 25% (i.e., 52 tissues) were removed from the dataset to serve as a validation set. The remaining 75% of the samples were used to train a model using twofold cross-validation.

Since the tonsil organs were sliced into 4 mm pieces and placed into one container for fixation, both the training and validation sets likely contain tissues from the same tonsil organ. Both sets of samples were unknown and randomized for the exact organ. The final model was tuned by selecting the number of valid components based on the percent of variance explained by each component as well as the mean squared predictive error. The final model was applied to the validation dataset to determine the accuracy of the model on blinded tissue spectra. A schematic flowchart of the model development is displayed in Supplementary Fig. S1.

### Calculation of MID-IR spectra

**FIG. 1.**
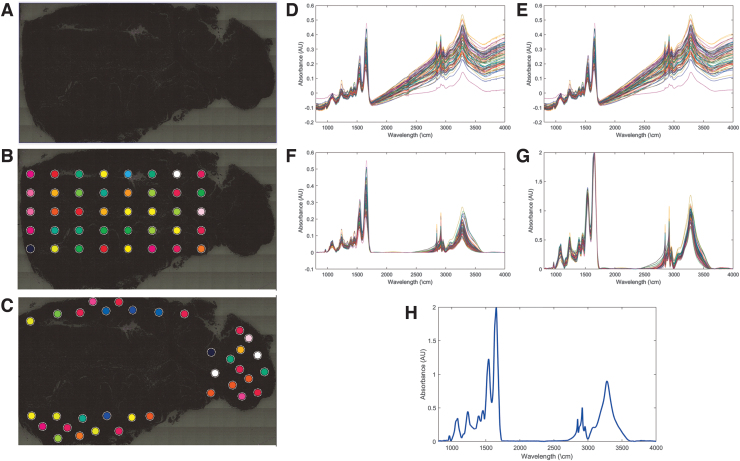
Overview of MID-IR collection. **(A)** Brightfield image of tissue sample acquired on Bruker Hyperion 3000 MID-IR microscope. **(B, C)** Regions of the sample that were spectroscopically imaged are indicated with the *colored circles*. **(D)** Original MID-IR spectrum of all points imaged within the tissue. Each line represents the spectrum of one *circle* in **(B, C)**. **(E)** MID-IR spectra after atmospheric correction. **(F)** MID-IR spectra after baseline correction to mitigate the effects of scattering. **(G)** MID-IR spectra after amplitude normalization. **(H)** Spatially averaged MID-IR spectra, representing the average MID-IR spectra of the entire tissue sample. MID-IR, mid infrared.

To correlate the MID-IR signature with known fixation times, we sectioned the same differentially fixed tissues onto MID-IR compatible slides (low-e MirrIR slides; Kevley Technologies) and captured the MID-IR signature using a Bruker Hyperion 3000 MID-IR microscope. Entire tissues were imaged using a visible low-magnification objective to coarsely map out the sample on the slide (Fig. 1A). Representative regions throughout the sample were then selected to be imaged using the mercury cadmium telluride single point detector (*ν* = 900–4000 cm^−1^, Δ*ν* = 8 cm^−1^, averages = 16). Approximately 100 regions located throughout each tissue were imaged to mitigate spatial heterogeneity in the MID-IR spectra (Fig. 1B, C).

Raw MID-IR spectra for each point are displayed in Figure 1D. These spectra were calculated by measuring the transmitted light, across all MID-IR frequencies, and dividing it by the transmission of the slide without tissue to get a measure of how much light the tissue was absorbing. Collected spectra were compensated for endogenous absorbers (e.g., CO_2_) present in the atmosphere that could impact the detected absorption spectra using algorithms in Bruker Optics Opus software (Fig. 1E). The baseline signal was then corrected to compensate for scattering within the tissue using a concave rubber band correction with 64 baseline points and 8–10 iterations (Fig. 1F). Each spectrum was then amplitude normalized to a global maximum (Fig. 1G), and average spectra from each tissue were calculated by averaging all the spectra from a given tissue together to calculate a high-quality representative spectrum for each sample (Fig. 1H). Spectral preprocessing was performed in Bruker Optics Opus software.

## Results

**FIG. 2.**
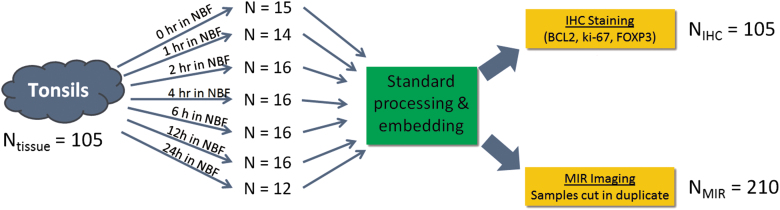
Graphical illustration of the design of experiment in which 105 individual pieces of tonsil tissue were differentially fixed in room temperature neutral buffered formalin for between 0 hour (i.e., unfixed/ethanol fixed) and 24 hours (fully fixed). Samples were equivalently processed through ethanol and xylene and embedded in paraffin. One slide from each tissue block was stained for BCL2, ki-67, and FOXP3 and two cuts from each block were imaged spectroscopically with the MID-IR microscope.

To study whether fixation status could be tracked via an MID-IR fingerprint, we designed a large-scale study with differentially fixed tonsil samples. One hundred five individual pieces of 4 mm thick human tonsil were differentially fixed for either 0, 1, 2, 4, 6, 12, or 24 hours in room temperature formalin (10% NBF). To account for intra-sample variability in the MID-IR spectra, each paraffin block was cut in duplicate and the 210 slides were imaged with an MID-IR microscope (Bruker Hyperion 3000 MID-IR). Between 12 and 16 tonsil samples were analyzed within each fixation time (Fig. 2 presents an overview of the experiment).

Since the samples were all equally processed, the only difference between the samples was the amount of time in formalin, that is, the chemical fixation time. Tissues in the study were stained with antibodies recognizing the proteins bcl-2, ki-67, and FOXP3. This enabled us to determine correlations between fixation time and functional staining. Biomarker expression was determined quantitatively by analyzing brightfield images with a developed digital quantitation program, as described later.

**FIG. 3.**
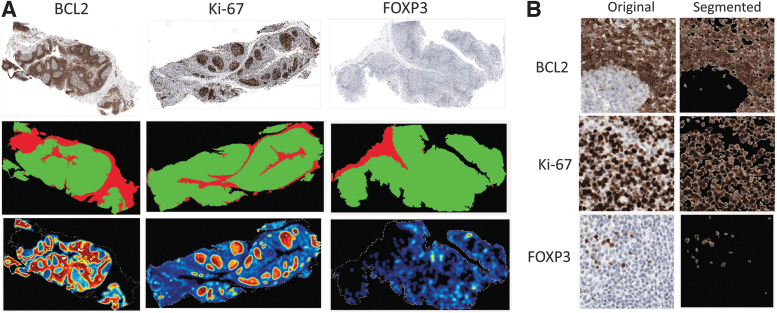
Overview of brightfield IHC imaging and image processing for biomarker quantitation. **(A)** Image segmentation algorithm. *Top row*: Original brightfield image of whole slide scans for the three antigens. *Middle row*: Results of image segmentation algorithm, *red* areas were excluded from digital analysis (stroma, connective tissue, etc.) and *green* regions were included in analysis. *Bottom row*: Hot spot rendering of biomarker expression level. *Red*, high expression density; *blue*, low expression density; *black*, negative/no tissue. **(B)** Example staining for all three antigens at 20 × including the original image (*left column*) and the segmented image (*right column*). IHC, immunohistochemistry.

We first needed to determine quantitative expression levels for each IHC stain, as a function of fixation time, to ultimately correlate a MID-IR signature with each tissue's fixation status. We separately developed an image analysis algorithm using brightfield images that segment the tissue on the slide and determine regions of the tissue that were not of interest (e.g., connective tissue, stroma) (Fig. 3A). The active regions of the tissue were then analyzed to determine whether the tissue was positive or negative for a given protein biomarker (Fig. 3A).

Heat maps were constructed for ease of interpretation. The data could be further developed into an overall percent positivity, representing the percent of the tissue's “active” region that was positive for a given antigen. Figure 3B shows a 20 × field of view images of representative regions of stain for each of the three markers and how the image analysis algorithm classified each.

**FIG. 4.**
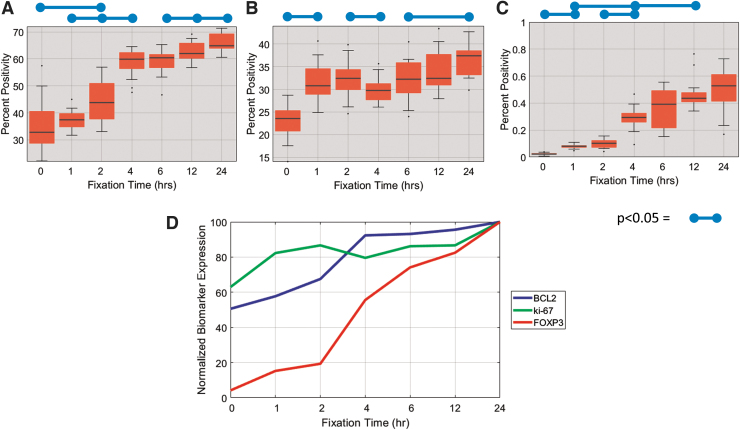
Quantitative analysis of IHC expression of **(A)** BCL2, **(B)** ki-67, and **(C)** FOXP3. **(D)** Plot of IHC expression for all three biomarkers versus fixation time in which the expression is plotted on a normalized scale so relative changes in each biomarker versus fixation time can be observed. Bars represent significant levels of *p* < 0.05 as determined by a double-sided rank sum test.

We analyzed 210 differentially fixed slides using the earlier described algorithm and correlated the results to the known fixation times of each sample to serve as a gold standard reference to fixation quality (Fig. 4). Results in the form of box and whisker plots versus fixation time are displayed in Figure 4A–C, for bcl-2, ki-67, and FOXP3, respectively. FOXP3 and bcl-2 were found to be particularly labile and susceptible to improper fixation, and their expression levels steadily increased with fixation time (Fig. 4A, C). Ki-67 was found to be robust to improper fixation as long as the biospecimen was fixed in NBF for at least 1 hour (Fig. 4B). Figure 4D displays the average expression level for each biomarker versus fixation time on a scale normalized to the maximum expression (i.e., 24 hours) for each respective biomarker.

Literature has suggested that the Amide I band in the MID-IR spectra is sensitive not only to the presence of protein absorbers but also to the confirmation state of the protein (e.g., β-sheet/α-Helix/Random Coil).^25–29^ Since formaldehyde forms covalent linkages with primary amines and causes conformational changes, we explicitly analyzed the MID-IR absorption of this spectral region, looking for deformations correlated with fixation time.

**FIG. 5.**
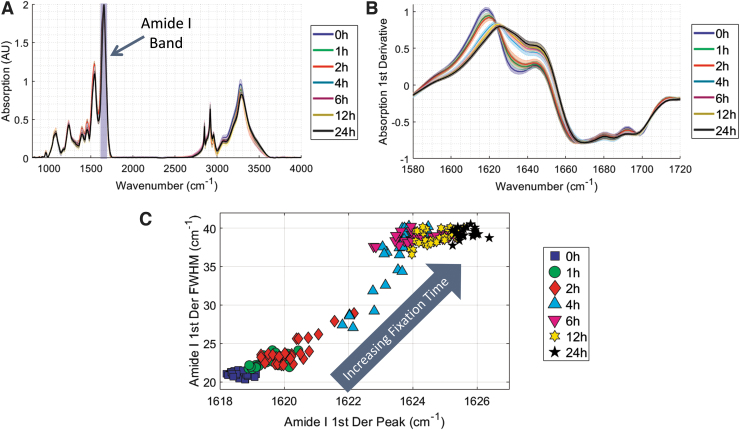
**(A)** Average MID-IR absorption for all fixation times, with the approximate location of the Amide I band indicated. **(B)** MID-IR absorption of the Amide I band. *Solid lines* represent average absorption, and error bars represent plus/minus standard deviation of all tissues. **(C)** Quantitative view of deformation of Amide I band in which the peak location of the band is plotted versus the full width half max.

The average MID-IR absorption for all samples for a given fixation time is displayed in Figure 5A, with the approximate location of the Amide I band indicated. The first derivative of each tissue's absorption spectra in the Amide I band was calculated using Savitzky-Golay differentiation to minimize noise in the calculation. The average first derivative with a standard deviation of variation, as indicated by the shaded regions, is plotted in Figure 5B. As fixation time increased, there was a clear shift to higher wavenumbers, the amplitude decreased, and the peak width increased.

These parameters of the band deformation are well characterized by the full width half max (FWHM) and peak location of the band, which are plotted in Figure 5C. There is a strong correlation between fixation time and Amide I deformation as 0 hour samples cluster in the bottom right and as fixation occurs the peak location shifts toward higher wavenumbers and the FWHM becomes wider. In this figure, the unfixed (i.e., 0 hours) and completely fixed (i.e., 24 hours) samples are clustered very tightly, indicating a high degree of reproducibility of the Amide I deformation metric. Interestingly, incompletely fixed samples (e.g., 1, 2, 4, 6 hours) tend to exhibit a more variable MID-IR signature, which highlights the sensitivity of the method to detect differences between partially fixed tissues.

**FIG. 6.**
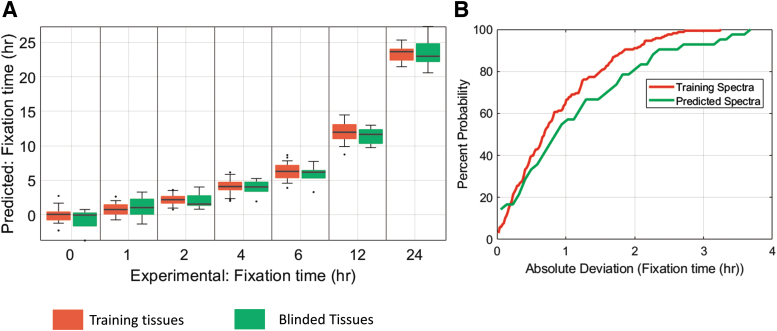
**(A)** Results of fixation prediction model on training data (*red*/*left*) and holdout blinded tissue samples (*green*/*right*). **(B)** Cumulative distribution function for the spectra from the training set and the holdout spectra. The developed model was able to predict the fixation time of unknown samples to 1.4 hours on average.

Previous experiments demonstrated that elements of the fixation process are manifest in the MID-IR spectra and show a coarse correlation between Amide I deformation and IHC status. To further those findings, we built a machine-learning model to search the entire spectra for a fixation fingerprint and used that model to quantitatively predict fixation times of unknown samples. The model was trained on 158 samples, and its performance was assessed on 52 validation samples. Results are plotted in Figure 6A.

Remarkably, across all fixation times, the model was able to predict the fixation time of unknown tissue with an average accuracy of 1.4 hours. These results indicate that the machine-learning model mined the true molecular fingerprint of formalin fixation and accurately predicted fixation times of unknown samples to roughly an hour, enough predictive power to be clinically relevant. The cumulative distribution functions for the fixation time predictions of the training as well as validation data are displayed in Figure 6B. The training and validation datasets have similar cumulative distribution functions, indicating that the model is not overfitting to noise or the underlying structure of the spectra in the training dataset.

**FIG. 7.**
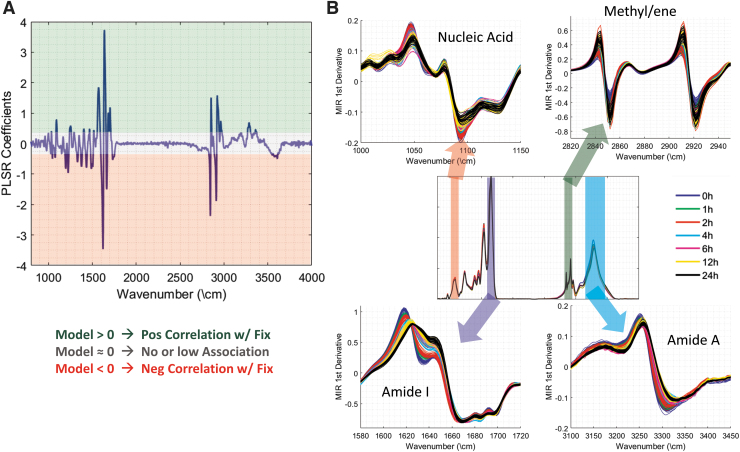
**(A)** Weights from developed PLSR model with positive coefficients representing a positive predictor of fixation time and negative coefficients representing a negative predictor of fixation time. **(B)** MID-IR spectra of four bands that showed differences correlated with fixation time. PLSR, partial least-squares regression.

One advantage of this approach is that the algorithm can be used to investigate the molecular fingerprint of fixation, as detected in the MID-IR absorption spectra. The model coefficients are plotted in Figure 7A, with values significantly greater than zero representing a frequency that is positively correlated with NBF fixation and values below zero representing wavenumbers that are associated with lack of fixation. As would be expected, there was a large contribution from the Amide I band near 1630 cm^−1^; however, several other important bands, such as Amide II and Amide A, also made contributions to the overall predictive power of the model. A number of these bands are plotted in Figure 7B to demonstrate that the developed model is using information throughout the wavelength range to make an accurate assessment of the fixation time.

To further test the ability of the MID-IR spectra to predict fixation status, we differentially fixed whole tonsils in a cold+warm fixation protocol that we have previously described.^23^ The cold formalin soak allows formalin penetration without crosslinking, whereas the second heated step drives rapid crosslinking of the previously diffused formalin. After each respective cold soak, each organ was soaked in warm formalin (45°C) for 1 hour to complete fixation. With this method, short cold soak times generate samples that contain well crosslinked edges and under-fixed interiors, whereas long cold soaks produce samples that are well fixed throughout the tonsil. By using whole tonsil organs, the areas of well-fixed and under-fixed interiors were more spatially pronounced, enabling the spatial profile of fixation to be assessed and visualized.

**FIG. 8.**
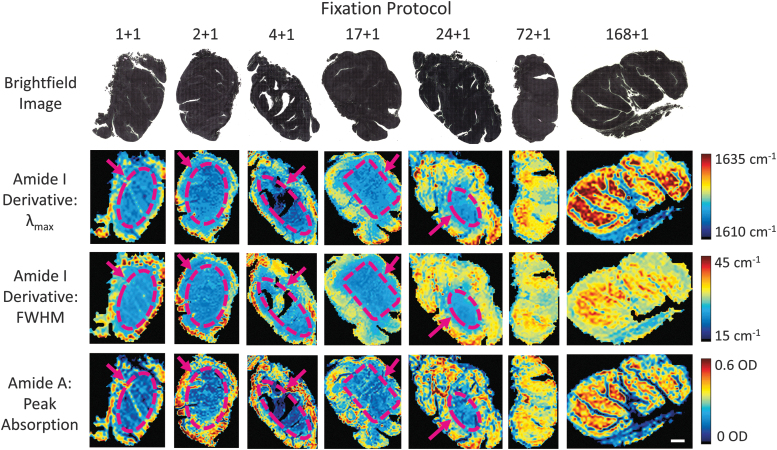
Two-dimension mapping of fixation signatures for variably fixed sections of whole tonsils. *Top row*: Brightfield images of unstained sections of whole tonsils. *Top-middle row:* Peak wavelength of the Amide I band's first derivative. *Bottom***-***middle row:* Full-width half-max of the Amide I band's first derivative. *Bottom row:* Peak absorption magnitude of the Amide A band. Central regions of tonsils that are underfixed according to MID-IR signatures are depicted with *purple dashed lines* and *arrows*. Scale bar = 2 mm.

We differentially fixed seven whole tonsil organs in cold 4°C formalin (1, 2, 4, 17, 24, 72, 168 hours) plus a 1 hour heating step to initiate crosslinking. A 4 mm section from the middle of the tonsils was removed and processed into wax blocks. For scale, brightfield images of the unstained section of whole tonsils are presented in Figure 8, top row. Spatial visualizations of several bands of the MID-IR spectrum for the differentially fixed tonsils are also presented in Figure 8 representing the peak wavelength of the Amide I band's first derivative (top-middle row), the FWHM of the Amide I band's first derivative (bottom-middle row), and the magnitude of the peak absorption of the Amide A band (bottom row).

Across all three signatures of the spectra, areas of poorly fixed tissues that are confined to the interior of each tissue can be visualized for fixation times of 24 hours or less (see dashed-purple regions). For the 72 hour and 168 hour samples, all three bands of the spectra have near-uniform expression, indicating that the tissue has been properly and homogeneously fixed for longer fixation times but not for shorter ones. This technique presents a powerful ability to analyze the spatial profile of a tissue's fixation quality. The spatial gradient in fixation quality was also shown to impact FOXP3 expression levels, in particular at the interior of each tissue (Supplementary Fig. S2).

## Discussion

We have demonstrated that fixation generates a significant and reproducible effect on a sample's vibrational spectra and can be used to determine a sample's formaldehyde signature. Further, we show in a large-scale study (>100) that the fixation time of a sample can be accurately determined to within, on average, 1.4 hours based on a trained machine-learning model. This finding was additionally validated with quantitative analysis of IHC staining for the biomarkers bcl-2, ki-67, and FOXP3, which confirmed the fixation quality of the tissue specimen. The sensitivity of the technique was demonstrated using larger sections from variably fixed specimens showing the accuracy and versatility of this predictive algorithm. This work establishes that an objective and standardized metrology to assess the quality of a tissue sample is possible using MID-IR spectroscopy coupled with a predictive machine-learning model.

This technology could provide a standardized and objective analytical method to ensure samples meet basic fixation requirements, even for samples with unknown fixation origins or from previously archived biobanks.

Tissues embedded into wax blocks are routinely used in medical applications and clinical trials. In many cases, analytical data regarding sample handling are missing or not provided. However, the largest error in pathology remains variable fixation due to non-standardized protocols. Qualitative methods are often employed by sectioning the block and performing an H&E stain to analyze the quality of morphological features. Trained pathologists evaluate samples and grade the quality. This is not always accurate, and it is very expensive to run histological stains with evaluation by highly trained pathologists. More routine standardized quantitative approaches would allow tissue sourcing to become quantitative, routine, and less costly, providing the highest quality tissue for experiments.

We have developed this technology based on the use of human tonsil samples, because they are easily obtained from a local medical facility, and they are a dense lymphatic tissue with a slow diffusion rate^30,31^ and a cellular composition where several IHC assays can be performed and monitored for fixation quality. Future experiments will need to verify the accuracy of this technology on several different types of medically relevant tissues, which we could not source in quantity. In addition, tonsil tissues used for these studies were from same-day patients, as would be the case in routine histology in medical centers.

However, there remains a large portion of tissues in wax blocks in tissue banks that have aged from days to many years. These banked tissue blocks are equally valuable for clinical assay and drug development. Similar experiments performed on aged tissue blocks would allow the MID-IR evaluation of this valuable resource.

## Supplementary Material

Supplemental data

Supplemental data
