# Supplementary material for: Assessing Tissue Fixation Time and Quality with Label-free Mid Infrared Spectroscopy and Machine Learning
Source: Biopreserv Biobank. 2023 Apr 17;21(2):208–16. doi: 10.1089/bio.2022.0108 (PMC10125394; doi:10.1089/bio.2022.0108)
Supplement: Supplemental data [file Supp_FigS1.docx]

^^[[1]](#footnote-1)^^

**Figure Legends**

**Figure s1.** Graphical depiction of how the PLSR model was trained.





1. [↑](#footnote-ref-1)
