# Supplementary material for: Assessing Tissue Fixation Time and Quality with Label-free Mid Infrared Spectroscopy and Machine Learning
Source: Biopreserv Biobank. 2023 Apr 17;21(2):208–16. doi: 10.1089/bio.2022.0108 (PMC10125394; doi:10.1089/bio.2022.0108)
Supplement: Supplemental data [file Supp_FigS2.docx]

**Figure s2.** FOXP3 expression plotted versus distance into tissue for differentially fixed whole tonsil samples. Longer fixation times resulted in increased staining, particularly in each sample’s interior.
